# EsoFLIP-assisted dilation for dysphagia in systemic sclerosis: Highlighting the role of multimodal esophageal evaluation

**DOI:** 10.1515/med-2025-1273

**Published:** 2025-09-09

**Authors:** Subhankar Chakraborty, Martha Yearsley

**Affiliations:** Department of Gastroenterology, Hepatology and Nutrition, The Ohio State University, 395 W 12th Avenue, Columbus, Ohio, 43016, United States; Department of Pathology, The Ohio State University, Columbus, Ohio, United States

**Keywords:** esophagitis, upper endoscopy, dysphagia, endoflip, systemic scleroderma

## Abstract

Scleroderma is an autoimmune disorder characterized by vasculopathy, progressive skin fibrosis, and dysfunction of internal organs. Esophageal involvement is seen in most scleroderma patients. There is no specific treatment for dysphagia caused by scleroderma. FLIP technology is a novel diagnostic and therapeutic modality to assess the tightness of esophageal sphincters and esophageal motility and also provide therapeutic dilation. We describe the case of a patient with scleroderma who presented with dysphagia. Upper endoscopy was unremarkable. Esophageal manometry revealed aperistalsis with normal upper and lower esophageal sphincter (LES) relaxation. Barium esophagram demonstrated poor esophageal motility and hold-up of the barium tablet at the gastroesophageal junction. EndoFLIP test during sedated upper endoscopy revealed normal LES distensibility and absent peristalsis of the esophageal body. Due to dysphagia, the LES was dilated with a 30 mm EsoFLIP. A barium esophagram following the dilation revealed a significant reduction in esophageal body diameter, normalization of barium tablet transit, and symptomatic improvement in dysphagia. Our case is the first report of the use of EsoFLIP to help improve dysphagia in a scleroderma patient. It illustrates the importance of multi-modal functional testing in patients with dysphagia and suggests the utility of EsoFLIP in the treatment of dysphagia in these patients.

## 1 Introduction

Systemic sclerosis (SSc) is a rare autoimmune disease characterized by progressive fibrosis with resulting dysfunction of multiple internal organs. The gastrointestinal (GI) system is frequently affected, with esophageal involvement being particularly common and often considered the classic GI manifestation of SSc [1]. It is estimated that esophageal involvement occurs in more than 90% of SSc patients [2]. Management of dysphagia in SSc patients is extremely challenging, relying on medications to control acid reflux, dietary modifications, and supplemental enteral nutrition through feeding tubes [3]. Prokinetics usually do not work well in SSc patients with dysphagia.

EndoFLIP is a novel diagnostic test for the evaluation of esophageal motility and sphincter compliance. In this technique, a catheter containing sensors that measure the diameter surrounded by a highly compliant balloon is positioned across the lower esophageal sphincter (LES). The balloon is progressively distended from 30 to 70 ml. The sensors give us the diameter of the sphincter in millimeters and pressure inside the balloon in mm Hg, and from that, the distensibility index is calculated as the ratio of the cross-sectional area and the pressure inside the balloon. Further, secondary esophageal peristalsis induced by distension of the LES gives us an idea of esophageal motility [4]. In a study comparing esophageal function in limited cutaneous scleroderma, diffuse cutaneous scleroderma, and normal controls using EndoFLIP, the main finding was that the cross-sectional area was higher in scleroderma patients than in controls. Among those with scleroderma, the cross-sectional area was the highest among those patients who had diffuse cutaneous involvement [5].

EsoFLIP is a form of hydraulic balloon dilation performed without the need for fluoroscopy using the FLIP machine. The balloon catheters go up to a maximum diameter of 20 or 30 mm. The 30 mm EsoFLIP balloon is used for esophageal dilation. It has been reported to be effective in patients with various causes of dysphagia, including achalasia and functional esophago-gastric outlet obstruction [6,7].

We present the case of a patient with SSc and dysphagia in whom a combination of techniques helped identify the cause of dysphagia. Treatment using the FLIP hydraulic balloon dilation system led to improvement in dysphagia and objective improvement in esophageal function. Our case is unique because it is the first report demonstrating the use of EsoFLIP technology to help dysphagia in scleroderma. It is also the first case to describe the distensibility characteristics of the upper GI tract sphincters in a patient with scleroderma.

## 2 Case presentation

A 56-year-old woman presented to the gastroenterology motility clinic to discuss her symptoms of acid reflux and constriction in the throat. She was referred to us by her rheumatologist to see if there were any new treatment options available for her. She had a history of scleroderma that was diagnosed 7 years ago. Two years back, she developed puffiness in her hands and developed Raynaud’s phenomenon. Additional medical problems included fibromyalgia, acquired hypothyroidism after surgery for multinodular goiter, irritable bowel syndrome with constipation, gastroesophageal reflux disease, obstructive sleep apnea, and chronic maxillary sinusitis.

In the clinic, she described severe difficulty with swallowing solids and pills, a sensation of food sticking to her throat, and odynophagia. Using a rating scale of 0–4, where 0 = no symptom and 4 = severe problem, her dysphagia score was 16. Food was getting stuck multiple times every day. She was also choking when eating food or drinking water, reported a globus sensation, took longer than 30 min to finish a meal, and reported unintentional weight loss of 27 lbs. in the last 6 months due to dysphagia. She reported rare globus sensation and chest pain, which was nearly always (90%) associated with food sticking in her throat after swallowing. She reported needing a balloon dilation previously, which had helped with dysphagia. She had heartburn 2–3 times every month, which persisted despite 30 mg lansoprazole twice daily for over 1 year. She also reported feelings of *postprandial* fullness, which often prevented her from finishing a meal. *Postprandial* regurgitation occurred 2–3 days per week, rarely accompanied by retching (10%), and often (50%) made her sick and vomit. It was sour tasting and rarely (less than 10% time) tasted pleasant. She also reported abdominal pain on most days of the week that worsened around the time of her bowel movements (80% of the time). Her stools were Bristol type 1 or 2 all the time. A review of her medications revealed that she was on nifedipine 30 mg twice a day, recommended for Raynaud’s phenomenon by her rheumatologist. She was not on opioids or other drugs that could affect esophageal motility. Her most recent labs were significant for normal hemoglobin, normal transaminases, and an elevated alkaline phosphatase (249 IU/L, normal range 32–126 IU/L). Nutritional markers, including vitamins A, B1, B12, B6, and D, niacin, serum iron, and total iron binding capacity, were normal. Iron saturation decreased to 13% (normal range 20–55%). She was recommended to start on a soluble fiber supplement and bisacodyl and complete motility testing.

**Figure 1:**
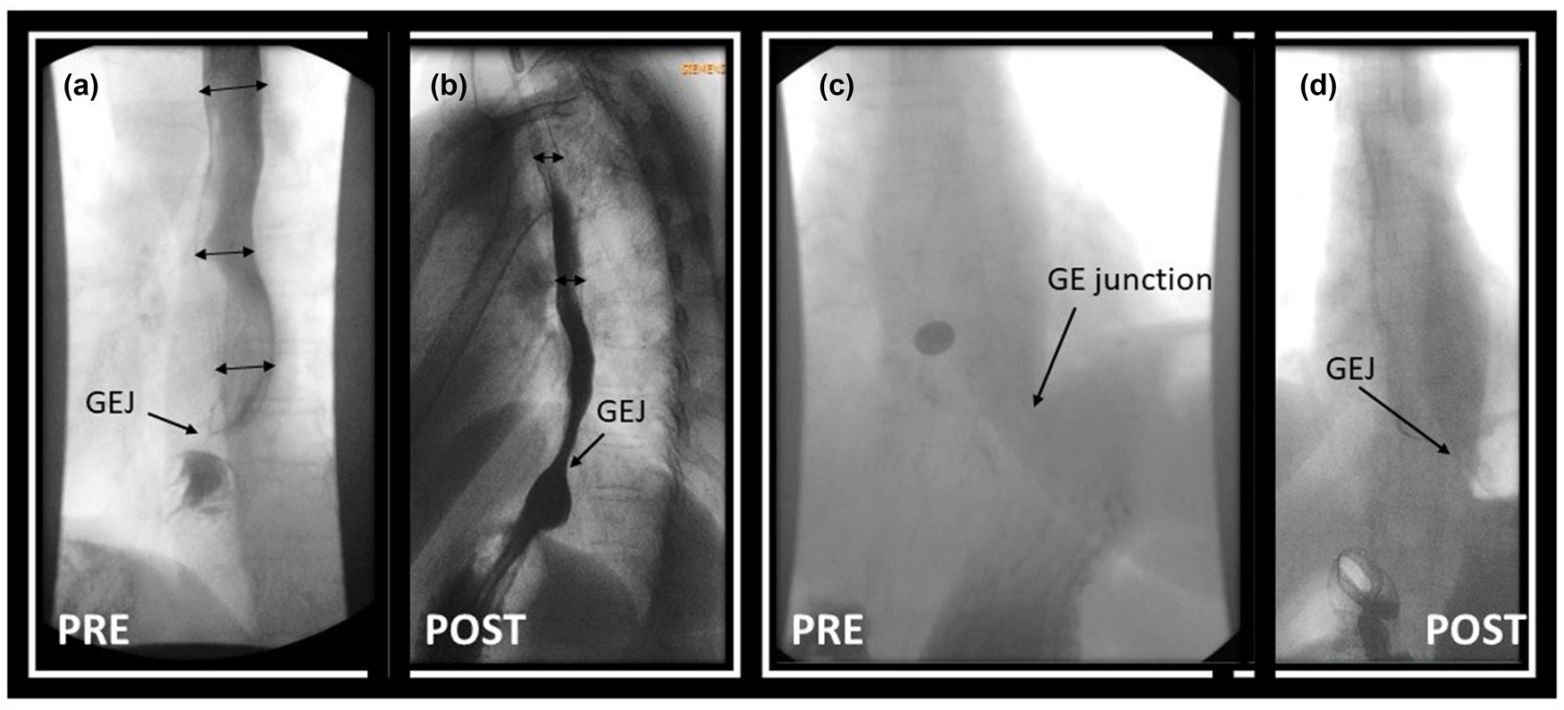
Esophagram with barium tablet. Dilated esophagus with stasis of liquids (a) and a 13 mm barium tablet stuck at the gastroesophageal (GE) junction (c). After dilating the GE junction with the 30 mm EsoFLIP, the esophagram demonstrates a decrease in esophageal diameter, improved clearance of liquids from the esophageal body (b), and normal passage of the barium tablet (d) into the stomach.

She underwent a barium esophagram test that revealed a dilated esophagus (Figure 1a), non-obstructing upper esophageal sphincter (UES), stasis of liquids in the mid esophagus, stasis of a 13 mm barium tablet at the gastroesophageal junction (GEJ) (Figure 1c), and a 2.5 cm hiatal hernia.

**Figure 2:**
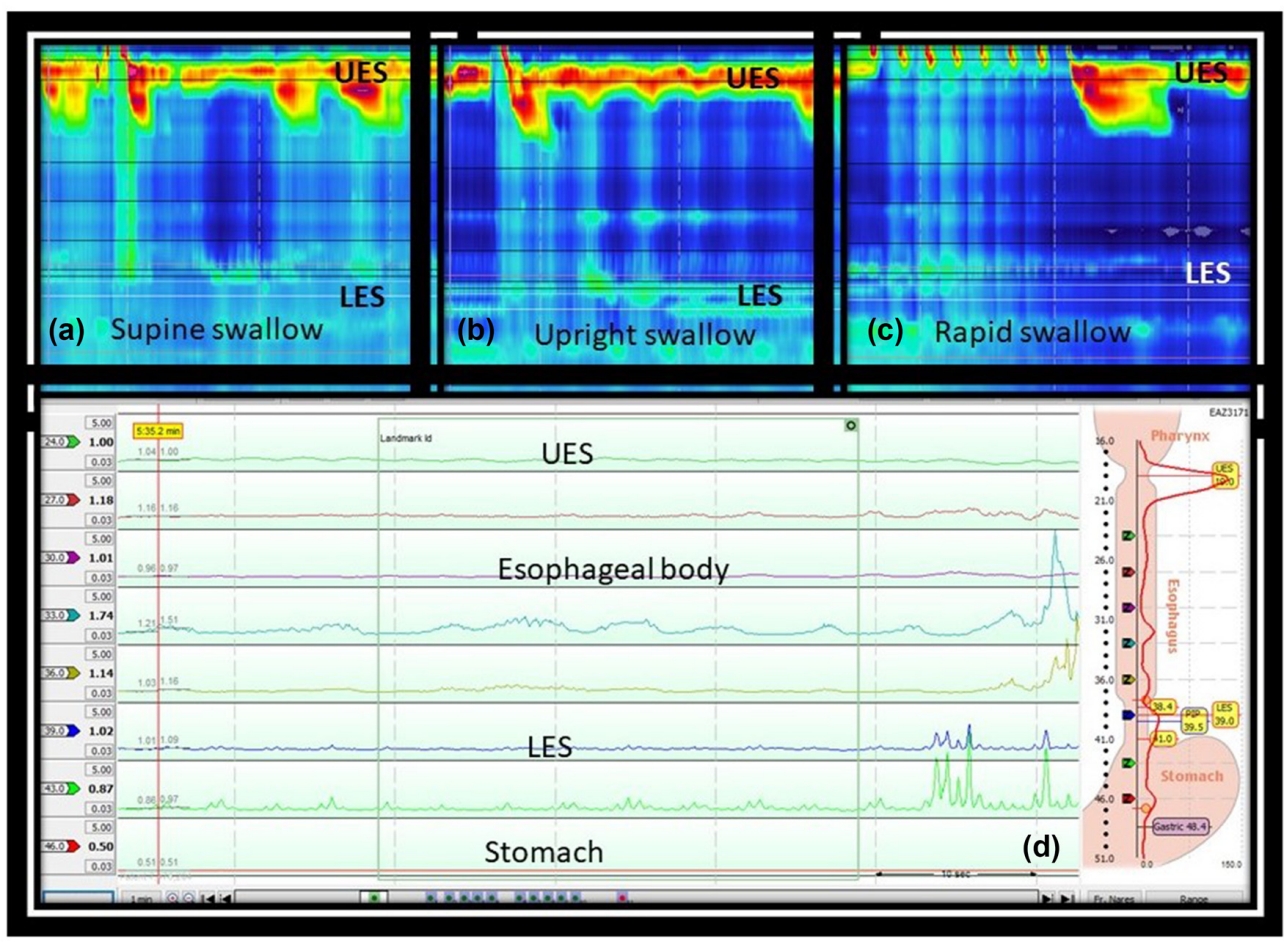
High-resolution esophageal manometry showing absent peristalsis with normal LES relaxation both in the supine (a) and upright (b) swallows. Multiple rapid swallow maneuvers demonstrate normal vagal inhibition during rapid swallow and absence of post-rapid swallow peristalsis (c). Baseline impedance analysis (d) revealed normal mucosal impedance (>1 kΩ) in the esophageal body.

High resolution esophageal manometry revealed 100% failed peristalsis in the esophageal body in both supine (Figure 2a) and upright position (Figure 2b). Normal response would be 100% intact peristalsis in both positions with normal LES pressure at rest and during swallowing (integrated relaxation pressure less than 15 mm Hg). Multiple rapid swallow maneuvers revealed normal vagal inhibition during rapid swallow, but absent post-swallow peristalsis (Figure 2c). Impedance analysis revealed normal mucosal impedance in the esophagus (Figure 2d). The LES pressure at rest was normal in the supine position but low in the upright position. LES relaxation, as assessed by the residual pressure, was normal. There was complete failure of peristalsis in both supine and upright swallows. The UES pressure at rest was high in the supine position, but it relaxed normally. None of the saline boluses were cleared completely from the esophagus, likely due to the failed peristalsis. Overall, the esophageal manometry test was suggestive of a connective tissue disorder.

A gastric emptying study was done given the symptoms of gastroparesis. It revealed 73% emptying after 1 h, 57% after 2 h, and 35% after 4 h, suggestive of delayed gastric emptying (normal study is less than 10% remaining after 4 h). She had previously tried metoclopramide without relief.

**Figure 3:**
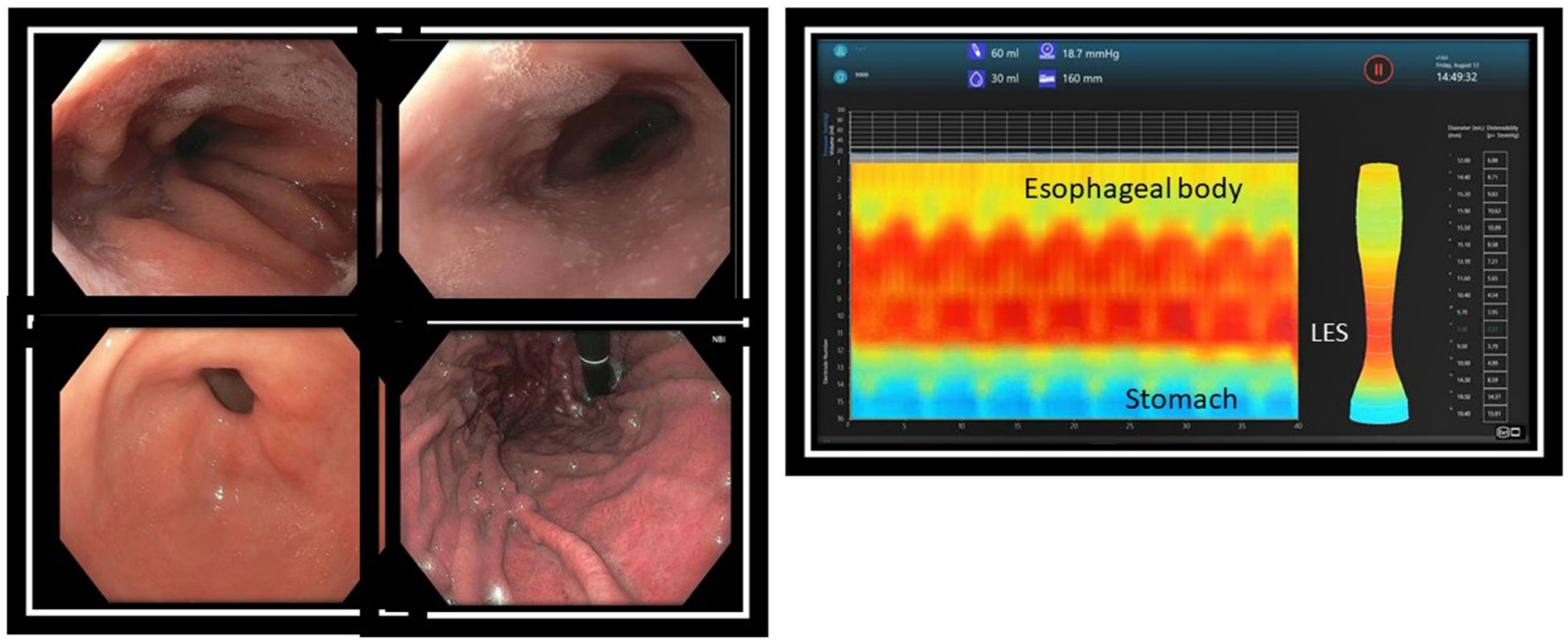
Upper endoscopy. Endoscopy revealed small white nummular lesions in the distal esophagus. Biopsies were positive for lichenoid esophagitis and *Candida* (left panel). EndoFLIP of the esophagus revealed a spastic pattern of esophageal motility without distinct propagated peristaltic activity (right panel).

Five months after the initial clinic visit, an upper endoscopy was done along with EndoFLIP under general anesthesia. Table 1 summarizes the EndoFLIP measurements at the lower esophageal, upper esophageal, and pyloric sphincters. Endoscopic examination revealed numerous white nummular lesions in the distal esophagus. Biopsies showed a lichenoid esophagitis pattern of injury with candidiasis (Figure 3).

**Table 1:** EndoFLIP measurements during initial upper endoscopy

Balloon volume	Distensibility index (mm^2^/mmHg)	Diameter (mm)	Intra-balloon pressure (mmHg)
**UES**			
30 ml	1.2	7.2	34.0
40 ml	3.2	12.7	39.0
50 ml	2.8	13.0	48.0
60 ml	2.4	14.0	64.0
70 ml	2.0	15.0	86.0
**LES**			
30 ml	1.0	5.5	19.0
40 ml	1.7	7.0	26.0
50 ml	3.2	10.0	28.0
60 ml	3.4	13.0	40.0
70 ml	2.7	13.0	53.0
**Pyloric sphincter**			
30 ml	4.6	11.0	21.0
40 ml	6.0	13.0	22.0
50 ml	5.6	15.0	31.0
60 ml	6.0	17.0	37.0
70 ml	4.2	18.0	61.0

**Figure 4:**
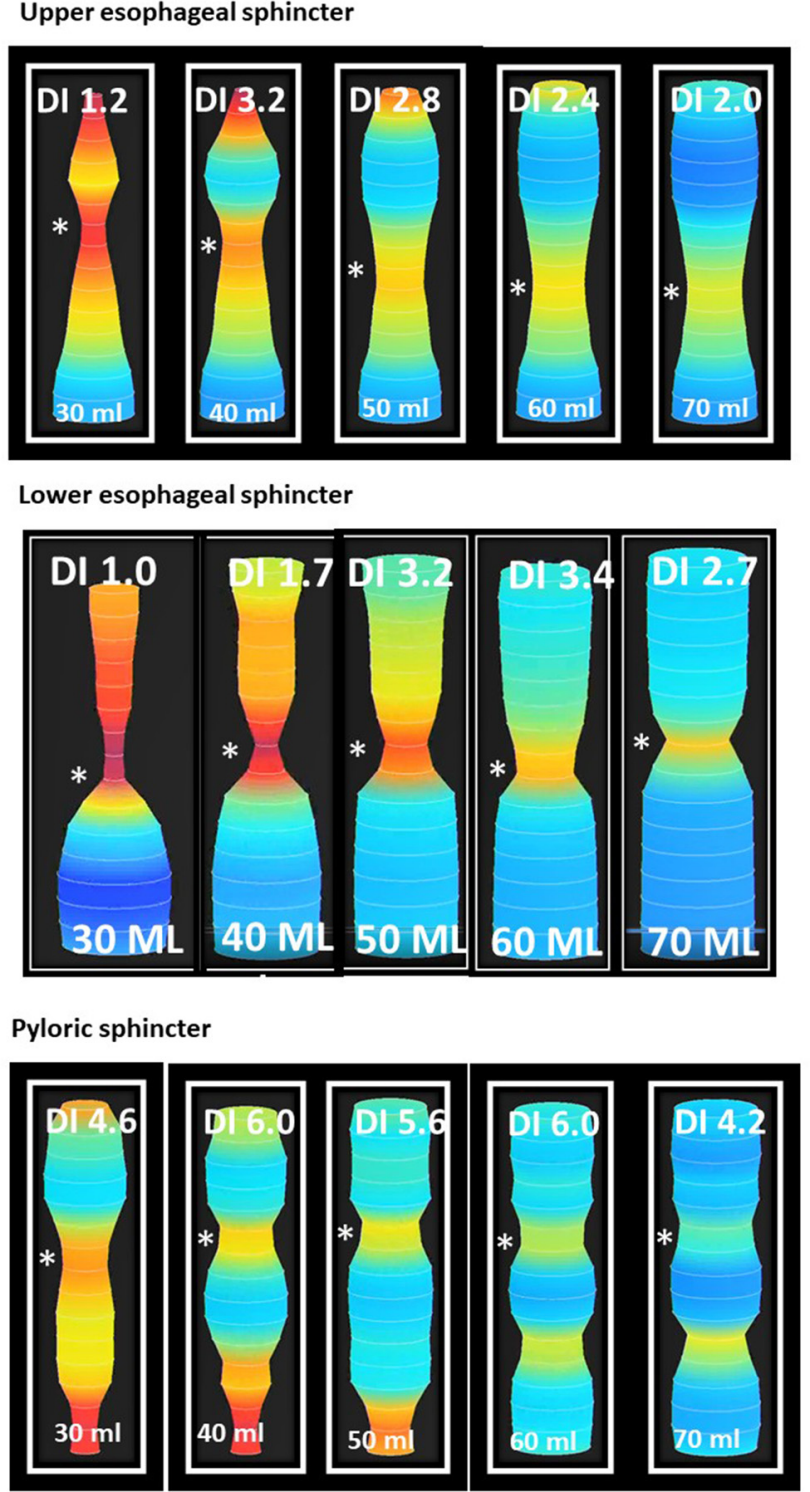
EndoFLIP assessment of the esophageal and pyloric sphincters. The figure shows pictures of EndoFLIP testing with distensibility indices of the UES (top), LES (middle), and pyloric sphincter (bottom). The volume of fluid inside the FLIP balloon is mentioned at the bottom of each panel in ml. The distensibility index is mentioned at the top of each panel in mm^2^/mmHg. The “*” symbol points to the location of the sphincter.

The EndoFLIP test was done using the EF-325 catheter. Figure 4 shows the response of the LES, UES, and pyloric sphincter to progressive distension. The results are summarized in Table 1. LES distensibility was normal (peak distensibility index [DI] was 3.4 mm^2^/mmHg, normal being >3 mm^2^/mmHg), UES distensibility was less than normal (peak distensibility being 3.2 mm^2^/mmHg, normal being >4 mm^2^/mmHg), and pyloric sphincter distensibility was reduced (peak distensibility being 6.0 mm^2^/mmHg with normal being >10 mm^2^/mmHg). There was a spastic pattern of esophageal peristalsis without any propagated peristaltic activity.

Due to complaints of a sense of constriction in the throat, a prominent cricopharyngeus on esophagram, and decreased UES distensibility on EndoFLIP, the UES was dilated up to 20 mm. The patient was prescribed a 3-week course of oral fluconazole to treat the esophageal candidiasis. Due to normal LES distensibility, we elected not to dilate the LES.

**Figure 5:**
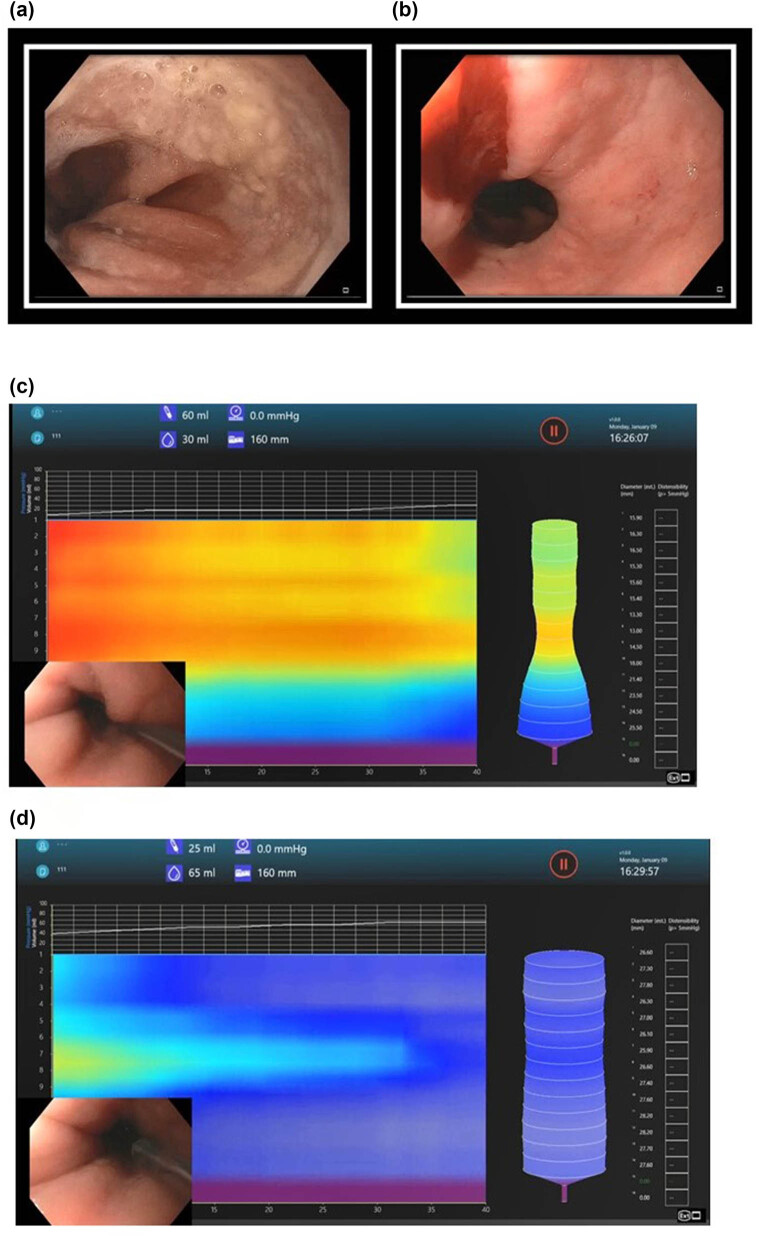
Second endoscopy for ongoing dysphagia 5 months after initial endoscopy. There were yellowish plaque-like lesions at the GEJ. Biopsies revealed lichenoid esophagitis without *Candida* (a). Mucosal bleeding after dilation of the GEJ with EsoFLIP (b). EsoFLIP balloon positioned across the GEJ with 30 ml of fluid in the balloon. This was used to measure the GE junction diameter before starting dilation (c). Maximum inflation leading to obliteration of the waist at the GEJ during EsoFLIP. The volume of fluid in the balloon was 65 ml. Inflation was held for 1 min at this volume before fully deflating the balloon (d).


When the patient followed up in clinic 2 weeks after the endoscopy, she reported no improvement in dysphagia despite dilation of the UES and treatment of candidiasis. She was already on lansoprazole 30 mg daily. Due to ongoing symptoms of dysphagia, hold-up of the barium tablet at the EGJ (Figure 1c), and lack of response to 20 mm CRE balloon dilation, an upper endoscopy with planned dilation using the EsoFLIP therapeutic balloon was performed 5 months after the first endoscopy. This time, there were yellowish lesions seen at the GEJ (Figure 5a). Biopsies again revealed a lichenoid-esophagitis pattern of injury but were negative for Candida. EndoFLIP showed normal LES distensibility (maximum DI 5 mm^2^/mmHg) with diminished peristalsis and improved UES distensibility (maximum DI 3.8 mm^2^/mmHg). Esophageal motility was still spastic. The LES and UES were now both dilated with a 30 mm EsoFLIP balloon (Figure 5c and d). There was mild mucosal disruption without perforation after the dilation (Figure 5b). The patient was discharged the same day after a 1 h observation period. There were no post-procedure adverse events or complications. After 1 month, the patient followed up in the clinic. She reported significant improvement in dysphagia. A follow-up esophagram done 6 months later revealed normalization of esophageal diameter as well as normal passage of liquids and barium tablet through the GEJ (Figure 1b and d). At a follow-up clinic visit after the esophagram, she reported occasional mid-chest pain, which was attributed to a 2.5 cm hiatal hernia seen on the esophagram. She rated her dysphagia symptoms as moderate in severity (score of 12), which was improved from that previously (score 16).

**Informed consent:** Informed consent has been obtained from the involved patient.

## 3 Discussion

SSc is an autoimmune connective tissue disease that results in dysfunctional collagen deposition in various internal organs, including any part of the GI tract. Esophageal involvement is very common. Symptoms in those with esophageal involvement are linked to impaired muscle function and typically include dysphagia, heartburn, and regurgitation [1,8,9]. Dysphagia, a distressing symptom in SSc, results from reduced or absent peristalsis and significantly impacts patients’ quality of life. Treatment is difficult and often requires placement of a feeding tube to maintain nutrition. Interestingly, a significant portion of patients, up to 40% in some studies, exhibit no symptoms despite documented esophageal motility issues by objective testing [8].

Esophageal dysmotility and reflux in the context of SSc can lead to substantial complications, including the development of esophageal strictures, Barrett’s esophagus, GI bleeding, and aspiration. The development of strictures in the esophagus is notably common and can stem from various factors, including acid reflux, damage from medications, and candidiasis. It has been estimated that SSc-related esophageal strictures may affect as many as 29% of patients [2,10].

Esophageal manometry is regarded as the definitive method for evaluating esophageal motility in individuals with SSc. Manometric abnormalities are detected in up to 90% of patients, even when they do not report symptoms. Typical findings on manometry include diminished peristalsis, especially in the lower part of the esophagus, and, in more advanced cases, aperistalsis along with reduced basal or resting pressure in the LES. Remarkably, the UES is typically unaffected, and contractility is maintained in the upper part of the esophagus [5,9,10,11].

The management of GI symptoms in SSc can be quite challenging. Treatments aimed at slowing or reversing the progression of SSc, such as high-dose immunosuppression and stem cell transplantation, have not shown significant effectiveness in addressing GI issues. Current therapies primarily focus on mitigating the complications arising from dysmotility. Initial interventions often include lifestyle modifications like raising the head of the bed, avoiding meals within 3 h of lying down, and steering clear of trigger foods. Currently, the mainstay of therapy for SSc-related reflux involves acid-suppressing medications known as proton pump inhibitors [10,12].

EndoFLIP is a novel technique that allows real-time, video assessment of esophageal sphincter distensibility and esophageal motility. It is performed during sedated upper endoscopy. It involves placing an 8 or 16 cm-long catheter with a special infinitely compliant balloon around sensors that measure diameter, cross-sectional area, and pressure. The balloon is positioned across the GEJ, and the balloon is usually inflated in 10 cc increments from 30 to 70 cc. The distensibility of sphincters is calculated as the ratio of the cross-sectional area to the pressure inside the balloon (mm^2^/mmHg). Normal LES distensibility is more than 3 mm^2^/mmHg. Smaller distensibility indicates a tighter sphincter and higher distensibility of a looser sphincter. Distention of the LES triggers secondary peristalsis in the esophageal body. This either moves from the mouth toward the stomach (called antegrade peristalsis), or from the esophagus toward the mouth (retrograde peristalsis). Sometimes, no peristalsis is seen (absent peristalsis), and sometimes, a mix of repetitive antegrade contractions and repetitive retrograde contractions may be seen (spastic peristalsis) [4,13]. While EndoFLIP has been reported in the assessment of a variety of esophageal disorders such as achalasia, GERD, and dysphagia, there is only one study that compared esophageal manometry and EndoFLIP in a small number (N-32) of patients with scleroderma. They classified the GE junction distensibility as reduced (DI < 2 mm^2^/mmHg, diameter less than 12 mm), borderline (<2 mm^2^/mmHg, maximum diameter <16 mm), or normal (≥2 mm^2^/mmHg and diameter ≥16 mm). Nearly 38% of scleroderma patients had absent contractility on EndoFLIP, 92% of whom also had absent contractility on esophageal manometry. In patients with absent contractility on EndoFLIP, the median EGJ-DI (8.8 mm^2^/mm Hg) was higher than in patients without absent contractility (5.5 mm^2^/mm Hg). There was no difference in maximum EGJ diameter (18 mm vs 19.7 mm), FLIP balloon pressure (20 mm Hg vs 38 mm Hg), presence of hiatal hernia (33% vs 60%), or the integrated relaxation pressure as determined by esophageal manometry [5]. We observed a discrepancy between EndoFLIP and barium esophagram findings in that there was a hold-up of the 13 mm barium tablet at the GE junction, while FLIP showed normal EGJ distensibility. There are several potential reasons. FLIP is performed under anesthesia, which relaxes the smooth muscle and thus may reduce any functional obstruction. Esophagram, by contrast, is performed while awake, which engages complex neuromuscular coordination and can reveal transient functional obstruction not apparent during a sedated FLIP. Further, FLIP measures distensibility, which is a measure of how easily the EGJ opens in response to balloon distension. Esophagram measures bolus transit, which depends on a combination of gravity, esophageal peristalsis, and relaxation of the EGJ. Distal esophageal spasm can impede the passage of the tablet even if the distensibility is normal on FLIP. The barium tablet requires both adequate relaxation and coordinated peristalsis to pass. FLIP, on the other hand, simply distends the lumen and measures its ability to open. While a patient may have a normally stretched EGJ, they may still lack coordination during awake swallowing. The barium tablet, being a solid, is more sensitive than FLIP to subtle mechanical resistance or delayed emptying at the EGJ. It may also be more likely to be impeded by a short-segment stricture or a mild Schatzki ring that may be missed on a FLIP.

EsoFLIP is a therapeutic dilation balloon used with the FLIP system. It comes in two sizes, with the 30 mm balloon holding up to 72 ml of fluid. This technique has been reported to improve dysphagia in patients with achalasia, esophagogastric junction obstruction, and post-fundoplication dysphagia [6,7]. The rationale to use EsoFLIP in this patient stemmed from failure of prior medical and endoscopic therapies, including CRE balloon dilation up to the maximum of 20 mm, and ongoing dysphagia, and was supported by a hold-up of the barium tablet at the EGJ on the initial esophagram, suggesting a functional EGJ outflow obstruction.

Lichenoid esophagitis pattern (LEP) refers to a histologic finding where there are features suggestive of lichen planus esophagitis, but where immunofluorescence was negative or was not performed. LEP is often seen in patients with infections like HIV, hepatitis B, hepatitis C, and hypothyroidism. Nearly 25% of patients have concomitant rheumatologic diseases such as rheumatoid arthritis, Raynaud’s phenomenon, fibromyalgia, lupus, and polymyalgia. In one study, nearly 62% of patients with LEP were taking more than three medications [14].

Our case highlights the importance of using multiple modalities rather than relying on a single one when investigating dysphagia. It also demonstrates how EndoFLIP can reveal the functioning of the three sphincters during upper endoscopy and can combine diagnosis with therapy. Our patient had no abnormality in the LES region on endoscopy, esophageal manometry, or EndoFLIP. It was the barium esophagram that gave us a clue that there may be a functional obstruction at the GE junction. Based on this and persistent dysphagia, the GEJ was dilated using the EsoFLIP balloon with subjective improvement in dysphagia, objective decrease in esophageal diameter, and improvement in esophageal emptying. We hypothesize that the combination of poor esophageal motility and functional GEJ obstruction was the cause of dysphagia in this patient.

Candidiasis can affect the esophagus in any immunosuppressed condition, including rheumatologic disorders like scleroderma. We often wonder whether it is the cause of the patient’s dysphagia. In this case, treatment of the infection led to the resolution of candidiasis but did not improve dysphagia. This is supported by a study from the Mayo Clinic, which found that candidiasis was very common in immunocompromised patients (43%) and its treatment did not lead to an improvement in dysphagia [15]. This suggests that sometimes it may simply be a bystander rather than the driver of dysphagia.

## 4 Conclusions

Scleroderma is a rare connective tissue disorder. Esophageal involvement is common in diffuse scleroderma and is a cause of significant morbidity. There are no FDA-approved therapies for dysphagia in scleroderma patients. EndoFLIP is a useful technique to assess the distensibility of the esophageal sphincters and secondary peristalsis. Our case highlights an example where manometry, esophagram, and EndoFLIP were discordant in a patient with scleroderma presenting with dysphagia. The decision to dilate the LES was based on the hold-up of a barium tablet on the esophagram and the symptoms of esophageal dysphagia. There was both objective and subjective improvement in dysphagia. Our case highlights the importance of using a multi-modality approach and considering symptoms along with test results when determining optimal therapy in patients with scleroderma and the utility of EsoFLIP in managing non-obstructive dysphagia.
